# Burden of Respiratory Syncytial Virus Hospitalizations in Canada

**DOI:** 10.1155/2017/4521302

**Published:** 2017-11-07

**Authors:** Ian Mitchell, Isabelle Defoy, ElizaBeth Grubb

**Affiliations:** ^1^Alberta Children's Hospital, 2888 Shaganappi Trail NW, Calgary, AB, Canada T6B 6A8; ^2^AbbVie Corporation, 8401 Trans-Canada Highway, Saint-Laurent, QC, Canada H4S 1Z1; ^3^AbbVie, Inc., 1 N. Waukegan Road, North Chicago, IL 60064, USA

## Abstract

**Objective:**

To examine the socioeconomic burden of respiratory syncytial virus (RSV) disease for Canadian infants hospitalized for the condition.

**Data and Methods:**

The descriptive study used data collected in Alberta, Canada, during 2 consecutive RSV seasons. Infants (<1 year of age) were included if they had not received palivizumab and were hospitalized with a confirmed diagnosis of RSV. Hospitalization resource use and parental time burden, out-of-pocket costs, lost work productivity, and stress and anxiety were assessed.

**Results:**

13.4% of all infants (*n* = 67) had intensive care unit (ICU) admission, and average ICU stay for these infants was 6.5 days. Families had average out-of-pocket expenses of 736.69 Canadian dollars (CAD $), and the average time both parents spent in hospital was nearly 7 days (164.0 hours). For working parents (*n* = 43), average absenteeism was 49% and overall work impairment was 77.8%. Parents also exhibited significant parental stress (3.6 on the Parental Stressor Scale: 43.9 state anxiety and 36.9 trait anxiety scores).

**Conclusions:**

Results indicate a high burden associated with the hospitalization of an infant due to RSV disease in terms of resource use, time, productivity, costs, and stress, even among a population of infants not considered to be at risk for the condition.

## 1. Introduction

Respiratory syncytial virus (RSV) is the most common cause of lower respiratory tract infections (LRTIs) in children worldwide [1] and is the leading cause of infant hospital admissions in developed countries [2, 3]. While nearly all children are affected by RSV by the time they are 2 years old [4], those less than 1 year of age have more severe RSV disease [5]. For example, in British Columbia between 2008 and 2010, 77% of all pediatric (<19 years of age) patients hospitalized with a confirmed diagnosis of RSV disease were age 1 or younger [6]. Overall, RSV was found to be a major viral cause of hospital admissions in Canada, with an estimated rate of 130 per 100,000 [7]. Infants with certain comorbid conditions, including prematurity, bronchopulmonary dysplasia, and congenital heart disease (CHD) are more likely to have severe RSV disease [8]. Only symptomatic treatment is available for these infants and may include a stay in the pediatric intensive care unit (ICU). While there is no effective vaccine, prophylaxis with a monoclonal antibody targeted at the RSV (palivizumab) may be administered by monthly injections throughout the RSV season. Palivizumab prophylaxis is generally reserved for vulnerable infants at the highest risk of severe disease [8].

Although only a small percentage of children in Canada require hospitalization for RSV [9, 10], a United States (US) study has indicated that inpatient care accounts for the majority of the total disease medical costs [11]. In addition to this evidence of high direct costs, studies from other countries have demonstrated that RSV hospitalizations are associated with significant indirect costs [12, 13]. Building on this previous research, the present study descriptively analyzed a sample of Canadian infants hospitalized with RSV disease with 2 goals. One of these goals was to identify possible risk factors for RSV hospitalization in Canada for patients under 1 year of age who were not eligible for and therefore did not receive palivizumab prophylaxis. The other was to provide a more complete and current picture of the socioeconomic burden of RSV hospitalization. This study examined both the direct costs associated with RSV hospitalizations and the indirect costs, including out-of-pocket (OOP) payments, lost productivity, and parental stress and anxiety.

## 2. Materials and Methods

This publication reports on data collected in Alberta Children's Hospital, Calgary, Alberta, Canada, for a study conducted during 2 consecutive RSV seasons (2010/2011 and 2011/2012). This was the only Canadian site in the Multinational Parent Burden Study, a prospective, observational survey-based analysis designed and implemented to quantify the humanistic burden that infant RSV/LRTI-associated hospitalizations have on parents and caregivers [14, 15].

Infants included in the study were < 1 year of age, had not received palivizumab prophylaxis, and were hospitalized with a confirmed diagnosis of RSV/LRTI, which was identified by a rapid direct fluorescent-antibody assay (DFA) test. Premature infants were 33–35 weeks of gestational age and were identified as having a low risk of RSV disease using the Canadian Risk-Scoring Tool (RST) [16]. The study was approved by the Conjoint Health Research Ethics Board, University of Calgary. Informed parental consent was obtained for all participants, and parent screening, consent, and enrollment occurred at the time of infant discharge. Parents completed a discharge survey and a follow-up survey, with data collected regarding parental characteristics, productivity, and parental stress and anxiety. Information on infant characteristics (severity of illness, comorbidities, and hospitalization) was obtained from chart reviews.

Measures of the burden associated with RSV were captured by hospitalization resource use and length of stay (LOS), as well as by the parental time burden and OOP costs associated with RSV hospitalizations (reported in Canadian dollars (CAD $)). In addition, lost work productivity was assessed via a Work Productivity and Activity Impairment questionnaire that was specific to and validated for caregivers of children hospitalized with respiratory illness (WPAI: CHRI) [17]. The WPAI: CHRI assessed absenteeism, presenteeism, overall work impairment, and activity impairment. Absenteeism was defined as time away from work, presenteeism was defined as impaired productivity while at work, overall work impairment was defined as a weighted sum of absenteeism and presenteeism, and activity impairment was defined as impairment in activities performed outside of work. WPAI: CHRI outcomes are expressed as impairment percentages, with higher numbers indicating greater impairment and less productivity [17].

The study also included patient-reported outcome (PRO) assessments of stress and anxiety for parents of infants with RSV. In addition to capturing resource utilization associated with infant RSV disease, stress levels were assessed by the Parental Stressor Scale: Infant Hospitalization (PSS: IH), while anxiety was assessed using the State-Trait Anxiety Inventory (STAI). The PSS: IH is a validated measure, scored on a 5-point scale that assesses stress experienced by parents when an infant is admitted to the hospital environment. The scale accounts for the stress induced by the appearance and behavior of the sick infant, the alterations in the parental role that occur as a result of the infant's hospitalization and treatment for acute illness, and the sights and sounds of the setting in which nursing occurs [18, 19]. The STAI comprises validated scales for state (S-anxiety) and trait (T-anxiety) anxiety. State anxiety evaluates past, present, and future feelings of apprehension, tension, nervousness, and worry and is an indicator of changes in transitory anxiety. The trait anxiety scale evaluates how people generally feel and determines feelings of anxiety as a long-standing general personality trait. Both the S-anxiety and T-anxiety scales are based upon the parent's responses to questions each of which requires a ranking from 1 to 4, with cumulative scores ranging from 20 to 80 and with higher scores indicating higher perceived levels of anxiety [20, 21].

This study was designed to describe the burden of the disease, with no a priori identified hypotheses. As such, there was no specific sample size required for the study. However, to obtain adequate data to inform results, the study set a goal of enrolling a minimum of 50 infants. All analyses were conducted using SAS, version 9.3 (SAS Institute, Inc., Cary, NC).

## 3. Results

Sixty-seven infants residing in Alberta, Canada (Table 1) met the study inclusion criteria. A slight majority were male (52.2% male versus 47.8% female), and a large majority were breastfed (86.6%), while about 1/5 (19.4%) were identified as having been born prematurely (mean gestational age = 34.5 weeks). With the exception of RSV, the infants appeared to be relatively healthy, with only a very small percentage having a comorbid diagnosis. The most frequently diagnosed comorbid conditions were chronic congenital heart disease (10.5%), a skin condition (6.0%), and chronic wheezing or asthma (4.5%). In a majority of the infant's families, the parents were married (97.0%) and had at least 1 child in day care (98.5%). A large proportion (43.3%) of the mothers (*n* = 67) and 29.5% of the fathers (*n* = 44) were self-identified as smokers (Table 2). Most of the parents in this study were employed, with 84.1% of the fathers and 9% of the mothers employed and currently working and with another 49.3% of the mothers employed but on parental leave.

**Figure 1:**
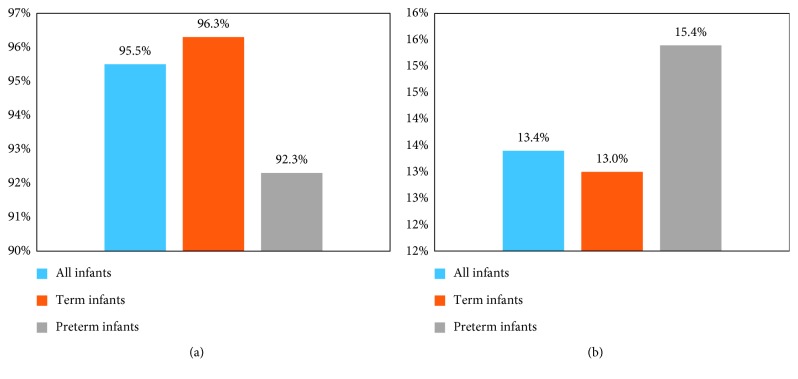
Healthcare resource utilization—by term status. (a) Use of supplemental oxygen. (b) Intensive care admission. Sample size: all infants (*N* = 67), term infants (*N* = 54), and preterm infants (*N* = 13).

**Figure 2:**
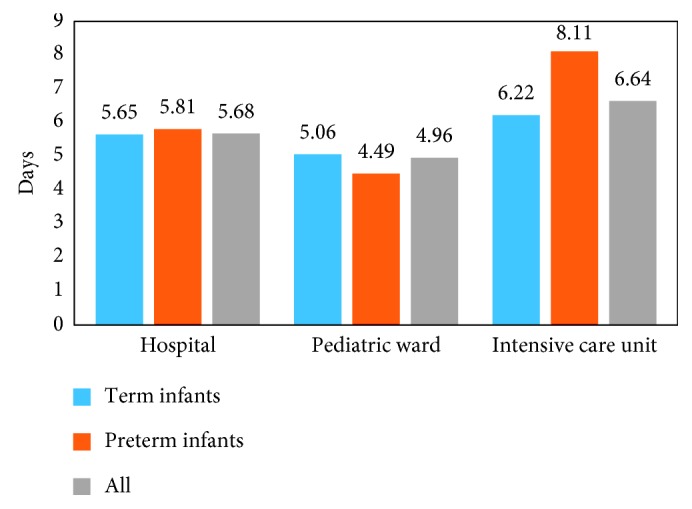
Hospital length of stay (LOS)—by term status. Sample size: hospital (*N* = 67): 54 term infants and 13 preterm infants. Pediatric ward (*N* = 66): 54 term infants and 12 preterm infants. Intensive care unit (*N* = 9): 7 term infants and 2 preterm infants.

Figures 1 and 2 illustrate the in-hospital resource use of all of the infants and compare the resource use of the premature and full-term infants. Results revealed that 95.5% of all infants received supplemental oxygen and 13.4% had an ICU admission. The average hospital LOS was 5.7 days overall and 5.1 days in the pediatric ward. Among patients who went to the ICU (*N* = 9), the average LOS was 6.5 days. While no tests of statistical significance were performed, results revealed that preterm infants were less likely than full-term infants to use supplemental oxygen (92.3% versus 96.3%) but more likely to have an ICU admission (15.4% versus 13.0%). The premature infants also had a longer mean ICU LOS (8.1 days versus 6.2 days).

In addition to hospital resource utilization, the study also gathered information about parental time lost, including work time, time at the hospital, and travel time to and from the hospital, as well as OOP costs associated with infant hospitalizations for RSV (Table 3). The average time both parents spent in the hospital was nearly 7 full days (164.0 hours), suggesting that each set of parents missed more than 3 days of work (24.7 hours/8 hours of work per day) when their infant was hospitalized. In addition to the time burden, the mean OOP expenses associated with an RSV hospitalization totaled CAD $736.69 and consisted of CAD $151.50 in transportation costs to/from the hospital, CAD $273.33 in costs for child care and home health, and CAD $311.86 in other OOP costs such as over-the-counter medications or medical equipment purchases, meals and lodging expenses, and any other nontransportation or home expenses, the parent classified as “other.” Parents of premature infants had lower transportation costs than parents of full-term infants (CAD $107.60 versus CAD $160.85), consistent with the hypothesis that their spending more time in the hospital was associated with fewer trips between the hospital and home.

**Figure 3:**
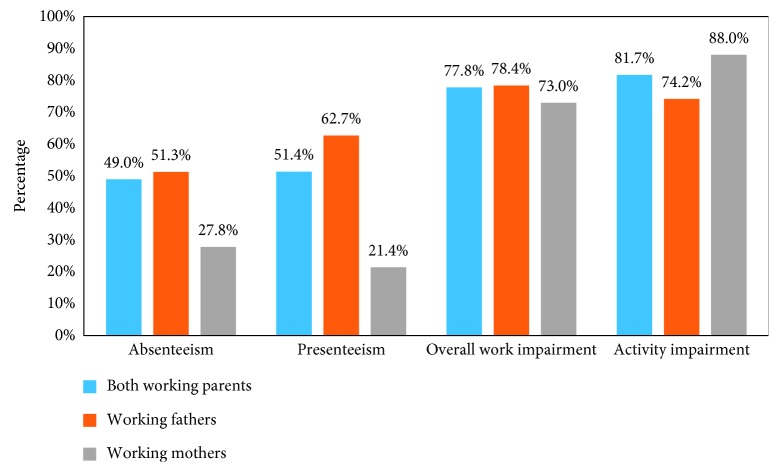
WPAI-CHRI. Sample size: both working parents (*N* = 43), working fathers (*N* = 37), and working mothers (*N* = 6).

**Figure 4:**
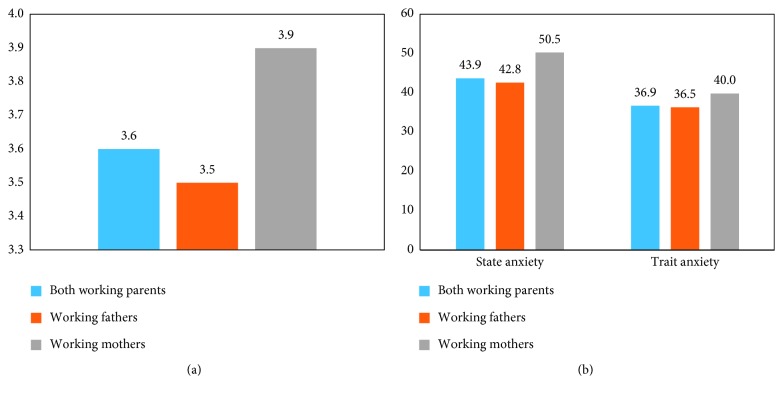
Parental stress and anxiety. (a) Parental stressor scale. (b) State-trait anxiety inventory. Sample size: both working parents (*N* = 43), working fathers (*N* = 37), and working mothers (*N* = 6).

Figure 3 illustrates the lost work productivity for both parents associated with their infant's hospitalization. Results revealed that average absenteeism was 49.0%, presenteeism was 51.4%, overall work impairment was 77.8%, and activity impairment was 81.7%. While working fathers experienced more absenteeism (51.3% versus 27.8%), presenteeism (62.7% versus 21.4%), and overall work impairment (78.4% versus 73.0%) compared to working mothers, working mothers reported more activity impairment than working fathers (88.0% versus 74.2%). In addition to lost work productivity experienced by working fathers and mothers, Figure 4 examines additional disease burden associated with the psychological health of the parents. The PRO instruments revealed that working parents, on average, scored 3.6 out of 5 on the Parental Stressor Scale, with mothers having a higher average score than fathers (3.9 versus 3.5). For all working parents, the average scores on the STAI were 43.9 S-anxiety and 36.9 T-anxiety, and as with the PSS: IH, working mothers had a higher score than working fathers (50.5 versus 42.8 for S-anxiety and 40.0 versus 36.5 for T-anxiety).

## 4. Discussion

This study examined the burden of hospitalization due to RSV disease for infants who were not considered to be at risk of RSV hospitalization. Results indicate that the burden is substantial in terms of hospital resources, parental costs, lost productivity, and psychological health. The following sections discuss and contextualize RSV risk factors and treatment costs revealed through this analysis of a population of infants residing across Canada.

### 4.1. Infant Characteristics

Consistent with a wide range of literature [22, 23], this study found a strong association between preterm birth and hospitalization for RSV. Specifically, preterm infants were overrepresented in this study, as they comprised 19.4% of the study population, compared to the estimated < 10% rate of preterm live births in Canada overall [24]. This overrepresentation of preterm infants was especially noteworthy given that, at the time the study was conducted, all pre-term infants < 32 weeks' gestational age, as well as all those who were 33–35 weeks' gestational age and identified as being at high risk of hospitalization, were protected from severe RSV disease with palivizumab and hence were ineligible for this study. Most of the children in the present study were < 6 months old, male, living with siblings, and either in day care themselves or the sibling of a child in day care, while a large minority had a mother (43.3%) and/or a father (29.5%) who smoked. These findings are consistent with a previous comprehensive review of the research, which reported these factors to be the ones most commonly associated with RSV hospitalization among both premature and full-term infants [23]. Finally, the majority (86.6%) of the infants in this study were breastfed, which is consistent with research which has identified breastfeeding as protective for RSV [23, 25, 26].

### 4.2. Hospital Resource Utilization

National healthcare statistics have shown that the largest share (29.2%) of Canadian healthcare spending is associated with hospitalizations [27]. Consistent with these data, the findings of the present study suggest that when children are hospitalized for RSV, they require inpatient resources at a very high rate. For instance, 13.4% of the infants were admitted to the ICU, with an average ICU LOS of nearly 1 week (mean ICU LOS = 6.5 days), while the preterm infants had even greater odds of an ICU admission, at 15.4%, as well as a higher mean ICU LOS of 8.1 days. Furthermore, the mean pediatric ward LOS in this study was 5.0 days, a finding roughly consistent with the Ontario Case Costing Initiative report of a mean hospital LOS of 3.91 days for pediatric RSV patients [28]. These observed associations between RSV hospitalization, ICU admission, and substantial ICU and hospital LOS are in concert with a number of previous studies, including a UK investigation which reported that RSV accounted for 15.6% of all admissions to intensive therapy units due to respiratory disease [29], a Mexican analysis which reported that 5.2% of infants hospitalized for RSV disease were admitted to the ICU [30], and a French examination which revealed that RSV patients in the pediatric ICU had a median ICU LOS of 8 days [31]. In addition to the significant direct costs, which have been reported in previous research [32], hospitalizations and ICU admissions for RSV disease represent a substantial humanistic burden. For instance, research has documented the dangers of pediatric hospitalization, with medication errors the most common adverse event [33]. In addition to medication errors, which have been shown to be associated with higher death rates in a pediatric population than in the adult population [34], pediatric hospitalizations are associated with other unique challenges, such as the inability of infant patients to convey information verbally to healthcare providers [35]. Moreover, the vast majority (95.5%) of the hospitalized infants in this study were given supplemental oxygen, likely based on pulse oximetry values. It has been shown that pulse oximetry use may lead to lengthening of the hospital stay [36], although acceptance of lower oximetry levels does not compromise the child's care [37].

### 4.3. Time Burden and Out-of-Pocket Costs

In addition to the burden on hospitals and patients, the families of the RSV-hospitalized infants in this study bore other substantial costs. These costs included the time spent visiting the child in the hospital and the travel time associated with such visits, as well as the related OOP costs for transportation, child care, and paid home help. On average, the family of each patient spent a total of nearly 1 week's time (6.8 days) in the hospital as well as 10.6 hours traveling to and from the hospital. Furthermore, these families incurred OOP costs for transportation (CAD $151.50), home costs (CAD $273.33), and other expenses, such as over-the-counter medications, medical equipment, meals, lodging, and other nontransportation or home costs (CAD $311.86). These findings are generally consistent with a previous investigation which reported that each RSV hospitalization in the US was associated with 218 hours of travel and visit time, as well as $827 (year 2000, US $) in average OOP costs, including travel, parking, visiting, meals, child care, and other expenses [13].

### 4.4. Work, Productivity, and Activity Impairment

Each set of parents in this study missed an average of 24.7 hours from work (absenteeism) because of their child's RSV hospitalization. It is hypothesized that this burden may be larger in other countries, such as the US, which do not have as generous a maternity leave policy as Alberta [38]. Furthermore, when a parent in the present investigation went to work during the hospitalization, he or she was, on average, 51.4% less productive than normal (presenteeism). Overall, parents reported an average work impairment of 77.8% due to RSV disease. The functional impairment of the parents also extended outside of work, as an average of 81.7% of each parent's regular, daily activities were prevented (activity impairment) due to the child's RSV hospitalization. Compared to previous reports from caregivers of older children with uncontrolled asthma [39], the parents of the infants with RSV disease in the present study reported much higher levels of absenteeism (49.0% versus 4.5%), presenteeism (51.4% versus 12.7%), overall work impairment (81.7% versus 15.9%), and activity impairment (78.4% versus 19.1%). While patients with uncontrolled asthma were significantly older than those in the current study and not all were hospitalized [39], the results appear to indicate a significant societal burden associated with RSV disease.

### 4.5. Parental Anxiety and Stress

The PSS: IH scale of parental stress showed that, in general, mothers experienced more stress than fathers (3.9 versus 3.5). This finding is consistent with research which examined premature infants in the neonatal ICU and found that mothers showed more trauma-related symptoms, were more stressed, anxious, depressed, and angry, and responded more negatively to the situation than fathers [40]. In contrast, other research which examined parents' stress both before and after congenital heart surgery, as well as a study of Chinese-American parents whose child spent 1–3 days in an ICU, found that fathers experienced more stress than mothers [41, 42]. However, both the mothers and the fathers of the infants hospitalized with RSV disease in this study had higher stress levels than did the parents in either of those earlier studies [41, 42]. For example, the examination of Chinese-American parents found average parental stress scores of 2.8 for mothers and 2.9 for fathers [42], compared to the average scores of 3.9 for mothers and 3.6 for fathers found in this study. Furthermore, the present study found a higher transitory stress level than that reported previously for mothers whose children were genetically at risk for type 1 diabetes (S-anxiety of 37.0) [43]. Given the large economic costs, marital instability, impaired functioning, and other adverse effects commonly linked to parental stress [44], the increased stress and anxiety of the parents in this study may represent additional economic and humanistic costs associated with RSV disease.

### 4.6. Limitations

This analysis was conducted using observational data collected from surveys of parents of infants with RSV disease in a pediatric hospital in Canada. Nonrandomization, recruitment bias, and the limited geographical catchment area are all factors that may have influenced the results and may therefore preclude generalization to broader populations. The limited geographical scope of the study is of particular note, since previous research has shown that RSV risk factors vary by region [23]. Further, given the small sample size, the study is purely descriptive in nature. As such, no comparisons between this cohort and a control group or between subgroups of infants, such as preterm and full term, were conducted. Further, the study is unable to statistically compare results across geographic regions and did not collect information on the use of palivizumab prophylaxis. Recall bias may have influenced the participants' responses regarding the amount of time spent away from work and the OOP costs incurred. However, much of the information was obtained at the time of infant discharge from hospital, and the PRO tools used in the study have been shown to have reasonably good psychometric properties [17, 18].

## 5. Conclusions

In conclusion, this descriptive analysis of Canadian survey data showed a number of key risk factors and a substantial burden associated with the hospitalizations of infants with RSV disease. Almost all (95%) of the hospitalized infants required supplemental oxygen, and 13.4% were admitted to the ICU. For those admitted to the ICU, the mean ICU LOS was approximately 1 week, while the mean pediatric ward LOS was also substantial, at approximately 5 days (mean). The adults who cared for these infants also had a significant time burden associated with visiting their child in the hospital, and they incurred additional burdens in terms of OOP expenses, absenteeism, presenteeism, daily impairment, and significant psychological stress. These findings extend the literature by analyzing the indirect costs of RSV hospitalizations in Canada, where parents will not face the additional burden of paying directly for physician care or costs of hospitalization.

## Figures and Tables

**Table 1:** Demographic characteristics of infants hospitalized and families of infants hospitalized.

Infants hospitalized (*n* = 67)		
Variable	Mean or *N*	SD or %
Sex		
Male	35	52.2%
Female	32	47.8%
Gestational age at birth, weeks (mean; SD)	37.3	1.7
Infant chronological age, months (mean; SD)	2.7	2.5
Breastfed		
Yes	58	86.6%
No	9	13.4%
Length of time child was breastfed, months^a^	1.8	1.8
Comorbidities^b^		
Allergies	2	3.0%
Chronic lung disease	1	1.5%
Chronic congenital heart disease	7	10.5%
Chronic wheezing or asthma	3	4.5%
Down's syndrome	2	3.0%
Severely immunocompromised	1	1.5%
Skin condition	4	6.0%
Maturity status		
Mature	54	80.6%
Premature	13	19.4%
Gestational age for premature infants, weeks (mean; SD)	34.5	2.1
Families of infants hospitalized (*n* = 67)		
Married		
Yes	65	97.0%
No	2	3.0%
At least 1 child in day care		
Yes	66	98.5%
No	1	1.5%
Number of children attending day care (mean; SD)	1.7	0.5
Number of siblings (mean; SD)	1.6	1.6

SD = standard deviation; ^a^among those who were breastfed; ^b^rates of cystic fibrosis and organ transplants were also examined although no infant received either diagnosis.

**Table 2:** Demographic characteristics of parents of infants hospitalized.

Variable	Fathers (*n* = 44)		Mothers (*n* = 67)	
	Mean or *N*	SD or %	Mean or *N*	SD or %
Age, years (mean; SD)	34.6	4.7	32.5	5.9
Education				
Degree/associate/bachelor/post grad/other	35	79.5%	48	71.6%
Attended some and/or completed high school	9	20.5%	19	28.4%
Employed status				
No	5	11.4%	28	41.8%
Parental leave	2	4.5%	33	49.3%
Yes	37	84.1%	6	9.0%
Smoker				
No	31	70.5%	38	56.7%
Yes	13	29.5%	29	43.3%

SD = standard deviation.

**Table 3:** Resource use and out-of-pocket costs associated with current hospitalization.

	All patients (*n* = 67)		Term infants (*n* = 54)		Premature infants (*n* = 13)	
	Mean	Range	Mean	Range	Mean	Range
Time burden during hospitalization (hours)						
Average time 1st parent spent in hospital	115.2	20–1,368	117.9	24.5–1,368	103.8	20–200
Average time 2nd parent spent in hospital	48.8	4–200	41.0	4–192	82.6	7–200
Parent time missed work during last 7 days	24.7	0–90	20.5	0–75	42.8	8–90
1st parent travel time to/from hospital	3.7	1–36	3.5	0.1–36	4.4	0.7–30
2nd parent travel time to/from hospital	6.9	2–30	7.6	2–30	3.9	3–8
Out-of-pocket costs associated with hospitalization (Canadian dollars)						
Transportation costs for 1st parent	55.26	2–400	56.76	5–400	50.60	2–80
Transportation costs for 2nd parent	96.24	10–760	104.09	10–760	57.00	20–79
Home costs						
Child care	185.83	25–700	203.00	25–700	100.00	100
Paid home health	87.50	75–100	87.50	75–100	—	—
Other out-of-pocket costs for 1st parent^a^	151.08	10–2,755	148.98	10–2,755	162.31	20–600
Other out-of-pocket costs for 2nd parent^a^	160.78	20–1,600	167.91	20–1,600	132.25	20–450

^a^Other out-of-pocket costs included costs for over-the-counter medications, medical equipment, meals, lodging, and any nontransportation or home costs listed by the parent as “other.”
